# Capturing Genetic Diversity in Seed Collections: An Empirical Study of Two Congeners with Contrasting Mating Systems

**DOI:** 10.3390/plants12030522

**Published:** 2023-01-23

**Authors:** Patricia Lu-Irving, Jason G. Bragg, Maurizio Rossetto, Kit King, Mitchell O’Brien, Marlien M. van der Merwe

**Affiliations:** 1 Research Centre for Ecosystem Resilience, Australian Institute of Botanical Science, Royal Botanic Gardens Sydney, Mrs Macquaries Rd., Sydney, NSW 2000, Australia; 2 Commonwealth Scientific and Industrial Research Organisation (CSIRO), Innovation Quarter Westmead, Level 3, East Tower, 158-164 Hawkesbury Rd., Westmead, NSW 2145, Australia

**Keywords:** germplasm, *Hakea*, inbreeding, outcrossing, selfing

## Abstract

Plant mating systems shape patterns of genetic diversity and impact the long-term success of populations. As such, they are relevant to the design of seed collections aiming to maximise genetic diversity (e.g., germplasm conservation, ecological restoration). However, for most species, little is known empirically about how variation in mating systems and genetic diversity is distributed. We investigated the relationship between genetic diversity and mating systems in two functionally similar, co-occurring species of *Hakea* (Proteaceae), and evaluated the extent to which genetic diversity was captured in seeds. We genotyped hundreds of seedlings and mother plants via DArTseq, and developed novel implementations of two approaches to inferring the mating system from SNP data. A striking contrast in patterns of genetic diversity between *H. sericea* and *H. teretifolia* was revealed, consistent with a contrast in their mating systems. While both species had mixed mating systems, *H. sericea* was found to be habitually selfing, while *H. teretifolia* more evenly employed both selfing and outcrossing. In both species, seed collection schemes maximised genetic diversity by increasing the number of maternal lines and sites sampled, but twice as many sites were needed for the selfing species to capture equivalent levels of genetic variation at a regional scale.

## 1. Introduction

In plant conservation and restoration, maximising genetic diversity in seed collections aims to support the persistence of re-established populations by promoting their adaptive potential [1,2,3]. In addition to enabling adaptive evolution in the long term, adequate genetic diversity can mitigate inbreeding depression as well as determine reproductive success in the short term, i.e., in outcrossing and self-incompatible species [4,5,6]. Plant mating systems shape patterns of genetic diversity among and within species and populations [7,8,9] but are often overlooked in the design of seed collections because they are poorly understood in most species.

The mating system is likely to be a major determinant of the extent to which seed collected under different strategies reflects the genetic diversity of standing populations. When mating systems are more selfing (vs. more outcrossing), it has been suggested that more effort is required to reliably capture genetic diversity both within populations [10] and among them (due to patterns in population structure; [11,12]). However, specific advice is sometimes lacking, particularly regarding the number of populations to sample to capture species-level genetic diversity [13], with general recommendations ranging from 5 sites to 50 [14,15,16,17] in order to meet the benchmark of a 95% probability of capturing all common alleles (>5% frequency) [10]. Empirical tests of the effect of the mating system on genetic diversity represented in seed collections are relatively few (notable examples include [18,19]).

While landscape-scale genomic data are increasingly applied to inform ecological restoration and conservation practice [20,21], there are few analytical tools for inferring the mating system from these data, and thus limited evidence of how variation in genetic diversity and mating systems are interrelated at this scale. In this study, we used recently described approaches [22] to characterise landscape-scale patterns in population genetics estimates, and developed new methods to infer patterns in mating systems. We tested whether similar species share similar patterns by comparing results from two congeneric, co-occurring, and ecologically similar species. Our goal was to provide empirical evidence to substantiate existing germplasm collection guidelines, and to support potential improvements to them. Specifically, we asked how the mating system might influence the genetic diversity captured by different approaches to seed collecting, and whether this might differ between the two species.

Data from genome-wide markers currently provides the most valuable proxy for understanding and capturing genetic variation for conservation purposes [23,24]. We genotyped thousands of Single Nucleotide Polymorphisms (SNPs) in hundreds of individuals of *Hakea sericea* and *Hakea teretifolia*, common shrubs of temperate Eastern Australia. We took a two-tiered approach to sampling. First, we sampled adult populations throughout the distributions of the species to characterise patterns of genetic diversity in the landscape. Then, we sampled wild plants and their progeny at a subset of sites to explore relationships between the genetic diversity, mating system, and the genetic diversity captured using different seed collecting approaches within and among sites. Tools to infer the mating system in incompletely sampled families from large SNP datasets are still emerging, so we used two approaches for estimating selfing rates from mother–seedling progeny arrays. The first was a reimplementation of Ritland’s [25] method of moments estimator (MME hereafter) for inferring individual single-locus selfing rates. The second is a novel approach to estimating individual levels of inbreeding based on a Convolutional Neural Network model (CNN hereafter). We compared selfing rates with patterns in heterozygosity, F-statistics, and relatedness, evaluating patterns in the context of seed collections for conservation and restoration.

## 2. Results

### 2.1. Genetic Diversity

#### 2.1.1. Landscape Genomic Patterns

**Figure 1:**
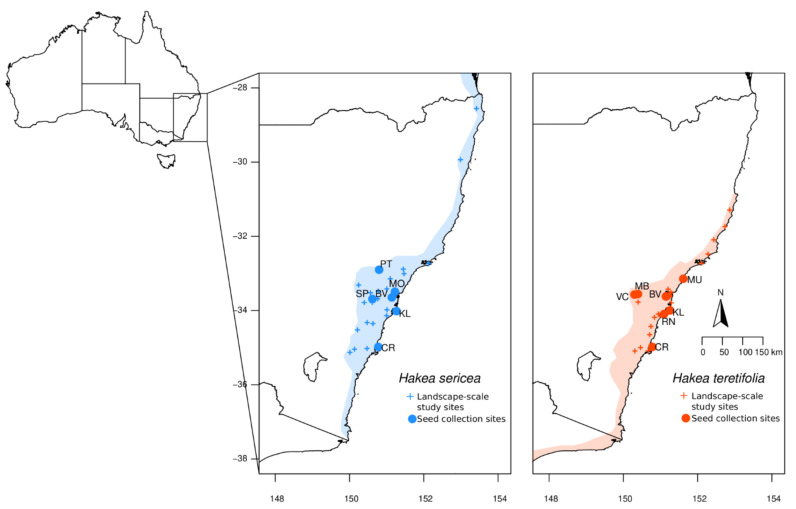
Maps showing distribution range of study species (shading), sites sampled for landscape-scale evaluation of genetic diversity (+), and sites sampled for progeny analysis (● and site code).

**Figure 2:**
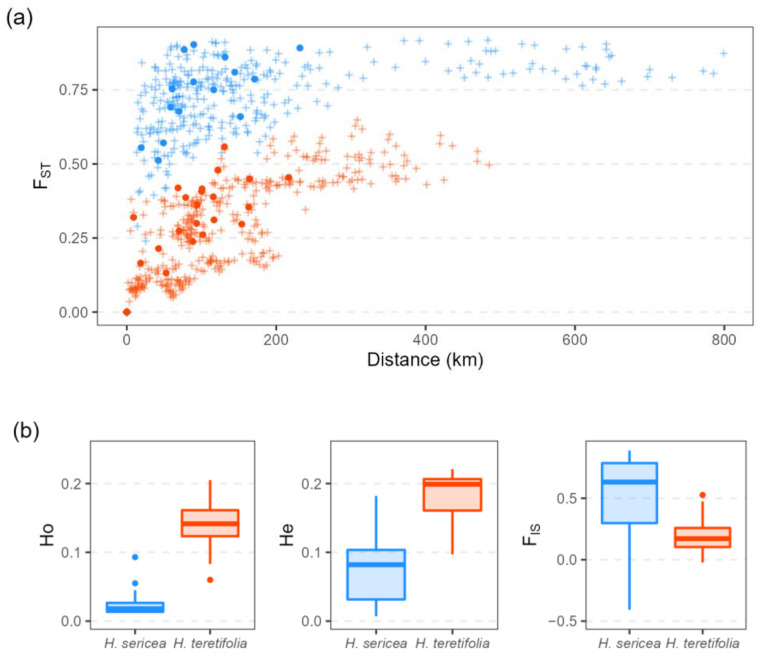
(**a**) Pairwise F_ST_ vs. geographic distance of *H. sericea* (blue) and *H. teretifolia* (orange) among populations sampled for landscape-scale evaluation of genetic diversity (+), and sites sampled for progeny analysis (●); (**b**) observed heterozygosity (Ho), expected heterozygosity (He), and F_IS_ estimates for populations sampled for landscape-scale evaluation of genetic diversity.

Analyses of broad-scale landscape genomic data revealed contrasting patterns between the two congeneric, co-distributed (Figure 1), and morphologically similar species. With a similar sampling strategy and sequence density, the DArTseq method captured SNP matrices of different sizes with 31,031 quality-filtered SNPs (from 48,728 raw SNPs) for *H. sericea* (203 total individuals across 31 sites) and 40,342 SNPs (from 80,527) for *H. teretifolia* (206 total individuals across 32 sites). Estimates of F_ST_ were higher in *H. sericea* (average 0.73) than in *H. teretifolia* (average 0.30), and estimates of observed (Ho) and expected heterozygosity (He) were lower in *H. sericea* (Ho average = 0.024; He average = 0.076) than in *H. teretifolia* (Ho average = 0.14; He average = 0.18; Figure 2). A positive relationship between F_ST_ and geographic distance was revealed among populations within both species, with a greater increase in F_ST_ over distance found in *H. sericea* compared with *H. teretifolia* (Figure 2a). We found a deficiency of heterozygotes in most populations of both species (observed heterozygosity was generally lower than expected under Hardy–Weinberg equilibrium), but six populations of *H. sericea* and three populations of *H. teretifolia* presented an excess of heterozygotes, resulting in negative F_IS_ estimates (Figure 2b).

#### 2.1.2. Mothers and Seedlings

A significantly higher overall germination success (proportion of total seeds germinated at the end of the experiment) was observed for *H. sericea* than *H. teretifolia* (94% vs. 52%, *p* = 1.01 × 10^−43^). The processed SNP data from seedling and mother samples comprised 19,287 SNPs for *H. sericea* (371 total individuals across 6 sites), and 35,899 SNPs for *H. teretifolia* (376 total individuals across 7 sites). Estimates of Ho, He, and F_IS_ of the seed mothers mostly fell within the range of population genomics estimates from the broader geographic sampling of wild plants (Figure 2b and Table S3), though some sites were consistent outliers (e.g., at *H. sericea* sites BV, MO in the Hawkesbury River valley, Ho and He were higher than at other sites).

### 2.2. Mating Systems

#### 2.2.1. Selfing Rate

The filtered data sets used for the estimation of selfing rates consisted of 5177 SNPs for *H. sericea* and 6990 SNPs for *H. teretifolia* following the exclusion of missing data. Methods for calculating selfing rate estimates ($\hat{s}$) using the MME and CNN approaches (hereafter ${\hat{s}}_{M}$ and ${\hat{s}}_{C}$, respectively), including validations and comparisons, are described in detail as Supporting Information. *H. sericea* site PT gave anomalous values (Figure S1) associated with high error (particularly in ${\hat{s}}_{C}$; Figure S2), possibly due to very low genetic variation at this site (Figure S3), and was thus excluded from further analyses of the mating system.

**Figure 3:**
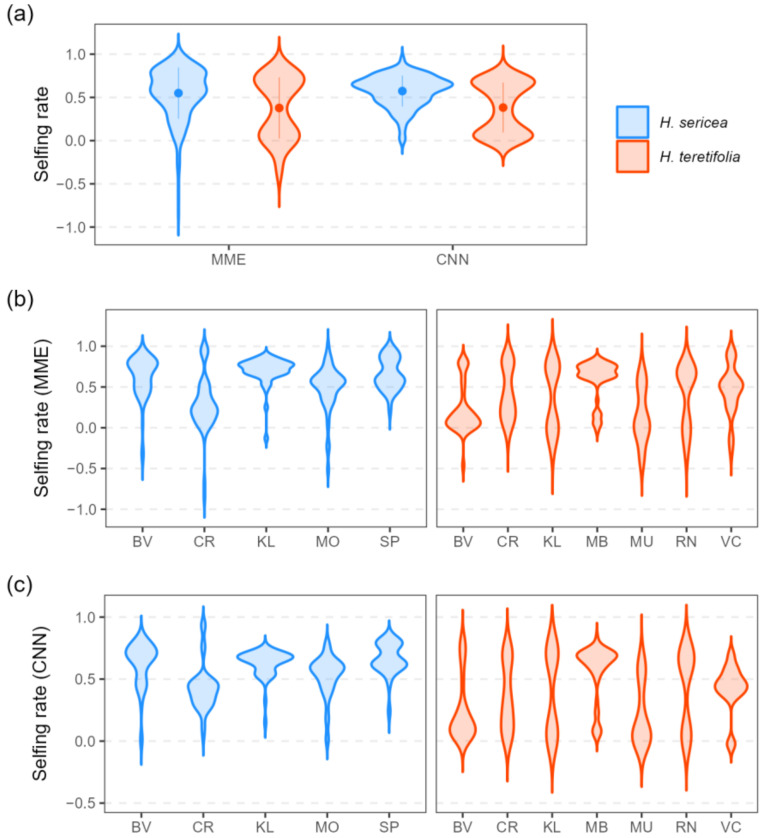
Violin plots (Gaussian kernel density estimates) of individual progeny selfing rates inferred using MME and CNN approaches for *H. sericea* (blue) and *H. teretifolia* (orange); per species (**a**), and per site (**b**,**c**).

**Figure 4:**
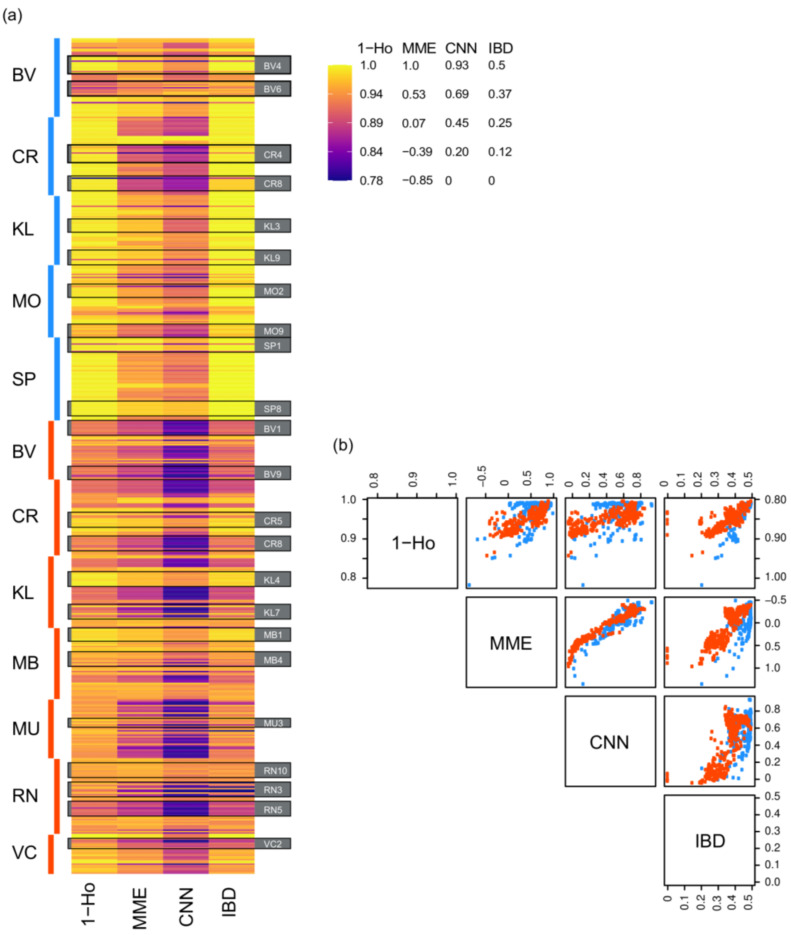
Comparisons among individual progeny of proportion of homozygous loci (1-Ho) and mating system estimates (MME: ${\hat{s}}_{M}$, CNN: ${\hat{s}}_{C}$, IBD: mother-progeny kinship coefficient, i.e., identity by descent). (**a**) Heatmap of scaled values side-by-side for each individual (horizontal stripe) grouped by species, site (vertical bar and 2-letter code), and maternal line (example maternal lines from each site are outlined and labeled with black boxes); (**b**) scatterplot matrix showing pairwise comparison of each set of individual values.

Estimated selfing rates were significantly higher on average in *H. sericea* compared with *H. teretifolia* using both MME and CNN approaches (${\hat{s}}_{M}$: *H. sericea* 0.55, *H. teretifolia* 0.38, *p* = 1.11 × 10^−8^; ${\hat{s}}_{C}$: *H. sericea* 0.57, *H. teretifolia* 0.38, *p* = 2.57 × 10^−12^; Figure 3a). Within each species, estimates of selfing rates varied among and within sites (Figure 3b); in *H. sericea*, ${\hat{s}}_{M}$ and ${\hat{s}}_{C}$ were on average lower in progeny from sites CR and MO compared with other sites, and in *H. teretifolia* ${\hat{s}}_{M}$ and ${\hat{s}}_{C}$, estimates were lower at sites MU and BV, and higher at MB. The range in estimates within sites and maternal lines also varied, in some cases as much as the range among sites (Figure 4).

The two methods for inferring the mating system developed in this study were robust under the validation conditions used (see Supporting Information) and yielded largely concordant results (Figure 3 and Figure 4). The means and ranges of ${\hat{s}}_{M}$ and ${\hat{s}}_{C}$ were similar among species and sites, but the shapes of the distributions differed. Distributions of selfing rate estimates within species and within sites were generally bimodal (tending to cluster around two modes), but the sizes and locations of the peaks varied among populations (Figure 3). At the species level, distributions of selfing rates had two distinct modes; in *H. sericea*, estimates were more frequently inferred to cluster around the higher selfing rate, whereas in *H. teretifolia*, values were more evenly distributed between a higher and a lower peak in selfing rate (Figure 3a). Similarly, clear bimodality was observed in estimates within most *H. teretifolia* sites, with relative sizes of peaks varying between sites (e.g., higher selfing rates more frequently inferred in site MB, and lower rates more frequently inferred in BV); however, peaks in distributions of estimates were less distinct within *H. sericea* sites (Figure 3b,c).

#### 2.2.2. Relatedness

Relatedness estimates were consistent with theoretically inferred values (i.e., ≥0.25 between parents and progeny, and ≥0.125 between siblings with unknown paternity), and were significantly higher in *H. sericea* than in *H. teretifolia* (Figure S4). Pairwise kinship coefficients between mothers and progeny showed a positive relationship with selfing rate (Figure 4).

#### 2.2.3. Patterns in Heterozygosity

Heterozygosity in the seed collection sites was lower in *H. sericea* relative to *H. teretifolia* (Table S3). There was broad concordance between the observed levels of heterozygosity and inferred mating systems; seedlings with relatively high proportions of heterozygous loci had lower selfing rate estimates (${\hat{s}}_{M}$ and ${\hat{s}}_{C}$), i.e., were inferred to be more likely to be outcrossed (Figure 4). Higher selfing was related to the loss of heterozygosity in seedlings relative to mothers, consistent with theoretical expectations (Figure S5); the difference in heterozygosity was negatively correlated with ${\hat{s}}_{M}$ and ${\hat{s}}_{C}$ in *H. teretifolia*, but in *H. sericea*, no significant correlation was found.

### 2.3. Effect of Sampling Strategy on Genetic Diversity in Subsamples of Seedlings

While sampling targets were the same among sites and between species, variation in germination success resulted in unequal numbers of seedlings being available for resampling. Germination success did not differ significantly among sites in *H. sericea*, but showed significant variation among *H. teretifolia* sites (e.g., CR 86%, MU and VC 18% and 20%, respectively; Table S2). Thus, there were too few *H. teretifolia* seedlings from sites MU and VC to meet resampling targets, and results for *H. teretifolia* are based on five sites (BV, CR, KL, MB, RN). Data were analysed from at least 9 maternal lines per site with at least 3 offspring each, and 3 maternal lines per site comprised at least 10 offspring (except *H. teretifolia* site BV, with only 2 maternal lines of at least 10 offspring; Table S1).

**Figure 5:**
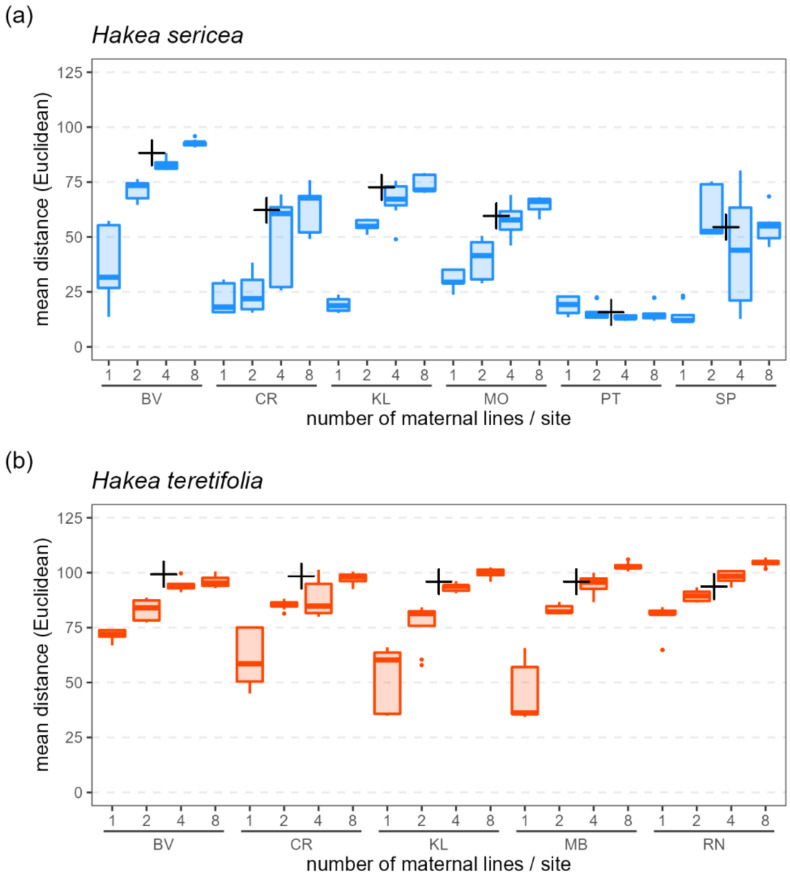
Genetic diversity (as average of pairwise Euclidean distances) among replicate subsamples of 8 seedlings per site for (**a**) *H. sericea* and (**b**) *H. teretifolia* according to 4 different sampling strategies (8 seedlings from 1 mother, 4 seedlings from 2 mothers, 2 seedlings from 4 mothers, 1 seedling from each of 8 mothers). Plotted for each site are averages among replicate sets of 8 seedlings randomly resampled 10 times per sampling scheme (boxplots), and average among the mother plants (+).

Within-site seedling subsamples which included more maternal lines had higher genetic diversity (within-subsample average distance) than those which included fewer maternal lines, in both species (Figure 5). At each site, targeting eight mothers resulted in the highest average genetic distances among offspring, whereas targeting only one mother resulted in the lowest (with the exception of *H. sericea* sites PT and SP; Figure 5).

**Figure 6:**
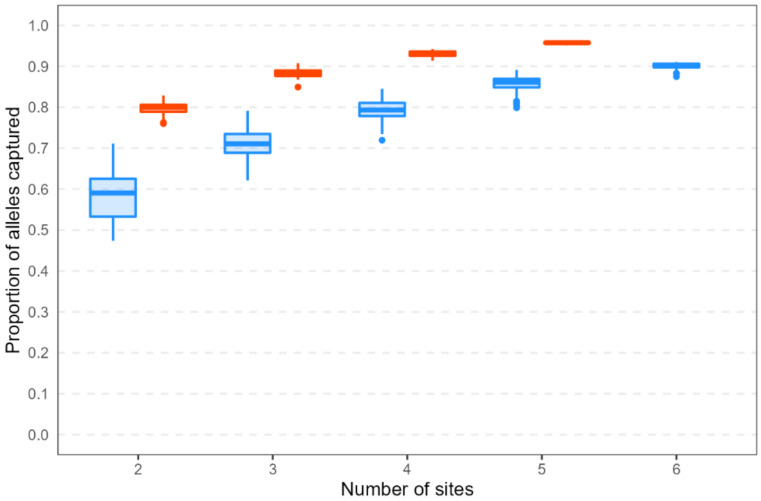
Proportions of alleles captured in seedling resampling replicates, out of all alleles found at ≥5% frequency in adult plants (i.e., including landscape-scale sampling sites from which no progeny were genotyped). Shown are results from 100 resampling replicates for 8 seedlings per site representing 8 maternal lines per site across increasing numbers of collection sites.

In both species, among-site seedling subsamples captured higher proportions of alleles when more sites were sampled (Figure 6). The maximum allele capture proportion increased as the minimum allele frequency threshold increased (i.e., as only increasingly common alleles were considered; Figure S6). For common alleles (those found on a landscape scale at ≥5% frequency), the proportion captured was lower, and the range in proportion captured was higher, among *H. sericea* resampling replicates compared with *H. teretifolia* (this pattern remained consistent even when the two most geographically distant *H. sericea* populations were removed; data not shown). In *H. sericea*, allele capture proportion ranged from 47% (2 sites) to 91% (6 sites). In *H. teretifolia*, allele capture proportion ranged from 76% (2 sites) to 96% (5 sites). No resampling replicate captured 100% of the common alleles in either species. Capturing 90% of the common alleles required at least 3 sites in *H. teretifolia* and at least 6 sites in *H. sericea*; capturing 80% of the common alleles required at least 2 sites in *H. teretifolia* and at least 4 sites in *H. sericea* (Figure 6).

## 3. Discussion

Empirical studies contribute towards understanding how genetic diversity is shaped within and among wild populations, and inform the development of practical, broadly applicable collecting guidelines to enhance the value of germplasm collections. Here, we gathered and analysed genomic data from two congeneric, seemingly functionally similar species, and developed tools enabling insight into mating systems using data from progeny analysis. This allowed comparisons exploring the extent to which population genetic patterns and reproductive behaviours are similar between related species (and among populations within species), as well as interpretation of the representative potential of seed collections and how to optimise these collections to best capture genetic diversity.

### 3.1. Contrasting Patterns of Genetic Diversity and Mating System in H. sericea vs. H. teretifolia

A clear contrast was revealed in the distribution of genetic diversity in the two species: low genetic diversity within populations (low heterozygosity, high F_IS_) and high differentiation among populations (high F_ST_) in *H. sericea* relative to *H. teretifolia*. Mating systems inferred for the two species from progeny analysis were consistent with the contrasting patterns in genetic diversity: while both species were capable of self-fertilisation and cross-fertilisation, prevalent selfing (and/or biparental inbreeding, discussed further below) was found in *H. sericea* (high selfing rate, high relatedness) relative to *H. teretifolia*. This relationship between patterns of genetic diversity and mating systems is consistent with expectations [8]. The strong contrast between the two species would not have been apparent without genetic data; *H. sericea* and *H. teretifolia* are similar in floral morphology and life history, and there are no prior reports on their mating systems (though variation in mating system among other species of *Hakea* has been recorded; [26,27]). While these contrasting patterns were unexpected given the outward similarities between *H. sericea* and *H. teretifolia*, they add to a growing body of findings demonstrating sometimes markedly contrasting patterns between species that are taxonomically related and otherwise functionally similar ([28,29]).

### 3.2. How Feasible Is It to Predict Mating System?

This study directly estimated selfing rates from progeny array datasets consisting of thousands of SNPs using novel methods (a reimplementation of a likelihood approach, and a Convolutional Neural Network). Results from the two approaches were concordant with one another under simulated scenarios, and with the empirical data, and were consistent with patterns of heterozygosity and estimated kinship coefficients, and we are optimistic that one or both of these approaches can extend to broader applications in the future.

The bimodality of selfing rate estimates is an intriguing pattern; one possible interpretation is that each peak corresponds to either outcrossed (lower ${\hat{s}}_{M}$ and ${\hat{s}}_{C}$) or inbred (higher ${\hat{s}}_{M}$ and ${\hat{s}}_{C}$) progeny. Anomalous ${\hat{s}}_{M}$ and ${\hat{s}}_{C}$ values from *H. sericea* site PT can be attributed to low heterozygosity together with exceptional genotypic uniformity leading to insufficient allele frequency variation for inference of selfing under expected rates of genotyping error. Possible explanations for this lack of diversity include multiple generations of inbreeding following a severe bottleneck (e.g., a single founder), and/or asexual reproduction (though we are unaware of prior reports of apomixis in *Hakea*); essentially, every plant sampled at this site belonged to a single isogenic (genetically identical) line. It is encouraging that error values associated with selfing estimates (particularly ${\hat{s}}_{C}$) suggested that inference for PT might be poorer than other populations.

While low selfing rate estimates indicate outcrossed seedlings (resulting from random mating within the population, e.g., in *H. sericea* site CR and H. teretifolia site BV), the specific process leading to high estimates can be harder to infer. When population heterozygosity levels are low and cross-fertilization occurs between close relatives, estimates of selfing rate can skew upward because biparentally inbred individuals can appear to be selfed [30,31,32]. Thus, while high ${\hat{s}}_{M}$ and ${\hat{s}}_{C}$ (such as in *H. sericea* generally and *H. teretifolia* site MB) are indicative of inbreeding, making the finer distinction between selfing and biparental inbreeding is difficult. We note, however, that this distinction is unlikely to impact seed sampling strategies for capturing genetic diversity at a site.

Consistent with theoretical expectations, species-level patterns of population genomic diversity (low heterozygosity, high F_IS_, and high F_ST_ in *H. sericea* relative to *H. teretifolia*) reflected a predominantly selfing and/or biparentally inbreeding mating system in *H. sericea* and a mixed mating system more evenly balanced between selfing and outcrossing in *H. teretifolia*. This suggests that, at a species level, the predominant mating system could be predictable from patterns of genetic diversity such as are increasingly commonly characterized in plant conservation and restoration studies (e.g., [22,33]). However, while patterns in F_IS_ (i.e., differences between observed and expected heterozygosity under Hardy–Weinberg equilibrium) were consistent with inferred mating system variation in this study, other factors (e.g., purifying selection) may also contribute. We note the sensitivity of F_IS_ to small sample sizes such as used in this study [34], and potentially also to genotyping error [35], especially when genetic variation is low (such as at site PT).

At the population level, patterns of genetic diversity did not always show expected relationships with the mating system. For example, at *H. sericea* site BV, heterozygosity and selfing rates were both relatively high, and F_IS_ was relatively low, compared to other populations of this species. Population demographic history and environmental variation (both spatial and temporal; [36]) are likely explanations for these patterns. While detailed exploration of such processes was beyond the scope of this study, we observed that the mating system varied widely among, and sometimes within, families, suggesting that any individual of either study species may readily produce both selfed and outcrossed seeds depending on intrinsic or extrinsic factors yet to be characterised. It is intriguing that inland populations of *H. sericea* (PT, SP) were presumably more highly inbred than coastal sites, but a similar pattern was not observed in *H. teretifolia*; additional study is required to test the influence of habitat on selfing rate. Our findings provide a foundation for further work using *H. sericea* and *H. teretifolia* as model systems to investigate the processes determining selfing vs. outcrossing in mixed-mating species. Future studies should focus on experimentation using increased within-population sampling for a subset of populations replicating particular environmental variables over time and space.

### 3.3. Genetic Diversity Captured in Seeds Reflects the Mating System of the Species

The within-site average pairwise genetic distance in subsamples of seedlings of both *H. sericea* and *H. teretifolia* increased as more maternal lines were sampled, and approached that of the wild mother plants when at least four maternal lines were sampled (Figure 5). We note, however, that the two *H. sericea* sites with the lowest observed heterozygosity did not follow this trend. Seedling subsamples from site PT lacked diversity regardless of the number of maternal lines represented, and seedling subsamples from site SP comprised greater genetic diversity when drawn from two or four maternal lines than eight. This effect is likely attributable to the mating system: repeated generations of selfing together with the fixation of alleles (e.g., via founder effects and/or selection) may result in a maternal line becoming so homozygous as to be essentially isogenic (genetically identical). Representing within-site genetic diversity is then a matter of distributing sampling effort to evenly capture all such lines present, noting that samples from multiple mother plants within the same isogenic line would be redundant for the purposes of capturing genetic diversity. Individuals from site PT comprised a single isogenic line (all were essentially genetically identical, and thus sampling from more mothers did not capture additional diversity in the seedlings), whereas site SP comprised four such lines (Figure S7).

Among sites, alleles found in the landscape-scale study of genetic diversity were better represented in resampled seedlings from the subset of seed collection sites in *H. teretifolia* than they were in *H. sericea*. In both species, the allele capture proportion increased as more sites were sampled, but the increase per site was greater in *H. sericea*. This reflects the distribution of genetic diversity within the two species, and is consistent with long-held expectations of the effects of mating system on population structure [8,11]. For regional-scale genetic diversity, the benchmark of capturing all common alleles (>5% frequency) with 95% probability [10] was not met by the subset of seed sampling sites in this study (five in *H. teretifolia*, six in *H. sericea*). Thus, our findings reinforce those of Neel and Cummings [13] in reporting that seed from five sites is insufficient to represent species-level genetic diversity, regardless of the mating system. Further, we showed empirically that it was necessary to sample more populations of a selfing species compared with a mixed mating species to maximise species-level genetic diversity in seed collections. More specifically, we found that twice the number of sites was required in the selfing species to reach the same proportion of allele capture as the mixed-mating species. Thus, we recommend that germplasm collections aiming to maximise species-level genetic diversity should double the number of populations sampled of a selfing species relative to an outcrossing or mixed-mating species.

The implications of highly structured population genetic diversity for making representative seed collections have been long understood, e.g., Brown and Marshall [37] suggested that among-site sampling should be prioritised in selfing species compared with outcrossing species, even at the cost of within-site sampling. However, this recommendation is explicitly restated in only two [16,38] out of six [3,17,39,40] recently published seed sourcing guidelines for germplasm conservation. In practice, there may be limited capacity to take mating systems into account (especially since mating systems are generally unknown for most species), which may explain the inconsistent dissemination of Brown and Marshall’s [37] advice. Ours is one of two recent studies explicitly testing and confirming this advice using empirical data: we inferred otherwise cryptic mating systems from SNP data, whereas Kallow et al. [19] had prior knowledge of the mating system. Given the concordance between patterns of genetic diversity and mating systems in both studies, and increasing trends towards gathering landscape-scale genomic data for plant conservation and restoration, the feasibility of predicting mating systems in data-deficient species should improve in the near future. Improved characterisation of mating systems should foster improved consideration of them as part of sourcing genetically representative germplasm collections.

### 3.4. How Does Mating System Modulate the Benefits of Genetic Diversity in Restored Populations?

We recommend that the causes and consequences of inbreeding (whether selfing or biparental inbreeding) be considered on a case-by-case basis. More specifically, we emphasize the distinction between a habitual inbreeder which has no significant loss of heterozygosity in selfed progeny and has presumably purged its genetic load at the population level (e.g., *H. sericea*), and a species with a more balanced reproductive strategy which loses heterozygosity when selfed and might suffer from the detrimental effects of excessive inbreeding (e.g., *H. teretifolia*).

In *H. sericea*, inbreeding depression might not pose much threat to population persistence, and populations might be more likely to establish from a smaller starting effective population size (Baker’s Law [41]; the success of *H. sericea* as an invasive species is consistent with this). Outbreeding depression [42] might warrant strategies to mitigate (e.g., local provenancing), on the other hand, maximising within-site diversity might secure greater resilience to change. While the long-term evolutionary consequences of habitual selfing as a reproductive strategy are likely to be negative [43], evolutionary timescales are usually beyond the scope of consideration when restoring populations from seed.

In contrast to the habitually selfing *H. sericea*, *H. teretifolia* might be more vulnerable to inbreeding depression; low germination rates at two *H. teretifolia* sites in this study could be a sign of this. We thus expect *H. teretifolia* to be an example of a mixed-mating species which will be negatively impacted by inbreeding, where it would be advisable to minimise relatedness when sourcing this species’ seeds for restoration and seed-banking practices. Furthermore, maintaining pollinator availability in restored populations of the mixed-mating *Hakea* species might be a priority to ensure their longer-term success [44], but could be considered less relevant in populations of selfing species such as *H. sericea*.

Our study is the first to our knowledge to use genome-wide marker data to empirically investigate strategies for capturing genetic diversity in seeds from a preferentially selfing species compared with its mixed-mating congener. Results confirm longstanding expectations by demonstrating that contrasting patterns of genetic diversity in a selfing compared with a mixed-mating species warrant contrasting collecting strategies (in terms of sampling more vs. fewer populations). Improved characterisation and consideration of mating systems in the design of germplasm collections will directly enhance the long-term viability of populations restored from such collections. Further studies focusing on the relationship between mating systems and genetic diversity in a germplasm conservation context should evaluate a varied range of species to ensure future seed-sourcing guidelines are broadly applicable.

## 4. Materials and Methods

### 4.1. Study Species

*Hakea sericea* Schrad. & J.C.Wendl and *Hakea teretifolia* (Salisb.) Britten (Proteaceae) are common shrubs of temperate woodlands and heath, endemic to southeastern Australia. These two species represent two clades in *Hakea* which radiated independently in southeastern Australia approximately 30 (±10) million years ago [45]. However, they share many morphological and functional traits, such as terete, needle-like leaves; small, white, zygomorphic flowers in axillary inflorescences; and woody, serotinous, two-seeded follicles (that of *H. sericea* is globose, 2–4 times thicker than the tapered follicle of *H. teretifolia*). Both species are serotinous, with a long-lived canopy seedbank and fire-enhanced dispersal, but are sensitive to high-intensity fires [46]. They can be found growing in sympatry on sandstone substrates in sclerophyll forests and heath where their distributions overlap (Figure 1). *Hakea sericea* is invasive in South Africa, New Zealand, and Europe.

### 4.2. Field Sampling

To characterise and compare landscape-scale patterns in population genomics within and between *H. sericea* and *H. teretifolia*, wild plants were sampled for genotyping across the distribution of each species in New South Wales, Australia (26 sites for *H. sericea* and 28 for *H. teretifolia*; Figure 1, Table S1a) following the restore and renew methodology for field sampling, DNA preservation, and genotyping (six individuals per site; [22]).

To explore mating system variation and the extent to which seed collections captured genetic diversity, we subsequently collected seeds and mother plant tissue samples from multiple sites for each species (six *H. sericea*: BV, CR, KL, MO, PT, SP; and seven *H. teretifolia*: BV, CR, KL, MB, MU, RN, VC; Figure 1, Table S1b). Sites sampled included coastal and inland populations representing the full elevational range of each species within the Sydney Basin Bioregion (Table S1b). At each site, leaf tissue was collected from 10 plants, each at least 10 m apart to minimise the sampling of first-degree relatives, and processed as described above. Multiple fruits were collected from each plant and stored in paper bags until seed extraction. Fruits were dried at 30 degrees C to open the follicles; extracted seeds were stored in paper envelopes under climate-controlled conditions until sowing.

### 4.3. Seed Germination and Seedling Sampling

Seed germination and seedling propagation were conducted at the Australian PlantBank. Seeds were surface sterilised by rinsing in 2% bleach for 3 min, then rinsing in autoclaved, deionised water, and immediately sown on 1% agar in petri dishes and incubated at a 12h/12h light/dark cycle. Seedlings were potted in forestry tubes, and two-to-three adult leaves were harvested from each seedling for DNA extractions and genotyping. Individuals were selected for genotyping to allow evaluation of the effect of sampling strategy on seedling genetic diversity relative to mothers: 10 maternal lines per site per species, with 7 represented by the mother and 3 progeny, and 3 represented by the mother and 10 progeny. This sampling was designed to enable replicate subsets of seedling data to represent a range of hypothetical seed sampling strategies, from few seeds/many mothers to many seeds/few mothers, within and among sites. When the number of available seedling tissue samples was insufficient to meet the target, all available samples were submitted (Table S2).

### 4.4. Genomic Data Collection and Processing

Lyophilised leaf tissues were randomized in 96-well tube racks and submitted to Diversity Arrays Technology Pty Ltd. (DArT) for DNA extraction and genotyping using the medium density DArTseq option. The DArT pipeline has successfully informed many studies across a wide range of model and non-model organisms (e.g., [47,48]) and is a cost- and time-efficient solution for generating genomic-scale data. Genotype data were provided as Single Nucleotide Polymorphism (SNP) matrices assembled through DArT’s proprietary analytical pipeline supplied with quality informing statistics for each bi-allelic marker and sample. In total, 1156 plants were genotyped using DArTseq.

Data were processed and downstream analyses were carried out using R. Prior to using the SNP data for downstream analysis, matrices were filtered based on several quality criteria using an in-house R package (see [22]). In summary, SNPs with a reproducibility score below 98% and missing genotype calls for more than 10% of samples were removed; to minimize the effect of linkage, only one SNP was maintained from markers with more than one SNP, and samples with more than 40% missing data were excluded from further analysis.

### 4.5. Estimates of Genetic Diversity and Relatedness

Genetic diversity measures were calculated from the filtered SNP data using the R packages diveRsity [49] (observed and expected heterozygosity, F_IS_) and SNPrelate [50] (population-pairwise F_ST_).

Pairwise kinship coefficients were estimated as probabilities that loci were identical by descent (IBD in the R package SNPrelate; [50]), using allele frequencies for each population estimated from the wild-sampled mother plants of that population (custom script). Kinship coefficients between species were compared using Mann–Whitney U tests.

### 4.6. Inference of Selfing Rates

We aimed to investigate mating systems in the context of seed collections for restoration and conservation, specifically asking how many of the progeny collected from 10 known mothers per site were produced via selfing vs. outcrossing. Several software tools exist for investigating mating systems, including MLTR ([25]; also see [51]) and COLONY ([52]). However, since MLTR was designed for microsatellite datasets (usually consisting of dozens to hundreds of markers each with multiple alleles), it was difficult to take advantage of our full SNP datasets (consisting of thousands of biallelic markers) using MLTR. The package COLONY usually relies on near-comprehensive population sampling, whereas we sampled individuals representing only a small fraction of the population at each site. We therefore developed tools for estimating the selfing rate (s) for each progeny that were suited to the available data. First, we implemented a single-locus method of moments estimator of selfing (MME) that was presented by Ritland [25]. We tested this implementation by generating progeny datasets that were simulated with different levels of selfing, but that reflected other features of the data, including the observed maternal genotypes and population allele frequencies, and estimated rates of genotyping error [53,54]. We checked that values inferred using the estimator were similar to those used in the corresponding simulations (see Supplementary Methods). Second, we performed more simulations of progeny data under different levels of selfing, and trained Convolutional Neural Network (CNN) models to predict rates of selfing. We then used the models to predict selfing rates for empirical progeny genotype data. Our goal was to develop an approach for mating system estimation that could be applied to many loci, and that, in the future, might be extended to data with different properties (e.g., with ordered markers) or to inferring different properties of mating systems (e.g., progeny produced by apomixis). Recent reports have shown that CNNs can be highly effective and flexible when applied to suitable genetic data and problems (e.g., [55,56,57]). Methods for calculating selfing rate estimates ($\hat{s}$) using the MME and CNN approaches (${\hat{s}}_{M}$ and ${\hat{s}}_{C}$, respectively), including validations and comparisons, are described in detail as Supporting Information (Supplement S1).

Statistical significance of differences in ${\hat{s}}_{M}$ and ${\hat{s}}_{C}$ between species was calculated using Mann–Whitney U tests. For each seedling, we then compared estimated selfing rates to mother–seedling difference in heterozygosity (proportion of heterozygous loci in seedling–proportion of heterozygous loci in mother) using Spearman’s correlation coefficient (rho).

### 4.7. Exploration of Seed Sampling Strategies

We used progeny array data to evaluate the range in genetic diversity captured by subsamples of seedlings according to different hypothetical sampling strategies (i.e., distributing sampling effort among fewer vs. more maternal lines and sites). We resampled replicate subsets of seedlings for each species by randomly drawing seedlings among maternal lines and sites according to the sampling schemes described below (custom scripts).

The within-site sampling schemes drew ten replicate subsamples of eight seedlings: one seedling from each of eight mother plants, or two seedlings from each of four mother plants, or four seedlings from each of two mother plants, or eight seedlings from a single mother plant. For each replicate subsample, pairwise Euclidean distance matrices were calculated and the mean of each matrix taken to express the genetic diversity captured per subsample per site. Euclidean distances were chosen to measure genetic diversity for their simplicity, lack of assumptions, and demonstrated concordance with other more complex metrics [58]; small within-site sample sizes together with unknown rates of genotyping error precluded the use of other metrics (such as counts of alleles captured).

Among-site sampling schemes drew 100 replicate seedling subsamples from among all seed collection sites per species. We compared the effect of sampling fewer vs. more sites with a scheme drawing one seedling from each of eight maternal lines per site, across increasing numbers of seed collection sites. For among-site subsamples, we evaluated allele capture by calculating whether both alleles at polymorphic loci in adult wild-sampled individuals were also present in each seedling subsample. We considered alleles occurring at ≥0.5%, 1%, 2%, and 5% frequency in mother plants (seed collection sites only), and all wild plants (landscape-scale study sites as well as seed collection sites). Alleles rarer than 0.5% were not considered due to the possibility of genotyping error in this frequency range.

## Data Availability

The data presented in this study are available as Supplementary Materials (Study Data). The code used for the inference of mating systems is accessible via a Zenodo repository: https://doi.org/10.5281/zenodo.7547080.

## Supplementary Materials

The following supporting information can be downloaded at: https://www.mdpi.com/article/10.3390/plants12030522/s1: Study Data; Supplementary Methods; Table S1: Sample collection sites and details; Table S2: Seed (progeny array) details and germination results; Table S3: Estimates of genetic diversity for seed sampling sites; Figure S1: Individual progeny selfing rates inferred per site (including site PT); Figure S2: Measures of error in selfing rate estimates; Figure S3: Genetic distance matrices among *H. sericea* individuals from sites PT and SP; Figure S4: Progeny array pairwise kinship coefficient estimates by known relationship; Figure S5: Loss of heterozygosity in progeny by selfing rate; Figure S6: Maximum proportions of alleles captured by resampling seedlings.
