# Supplementary material for: Capturing Genetic Diversity in Seed Collections: An Empirical Study of Two Congeners with Contrasting Mating Systems
Source: Plants (Basel). 2023 Jan 23;12(3):522. doi: 10.3390/plants12030522 (PMC9921034; doi:10.3390/plants12030522)
Supplement: Supplementary file 1 [file plants-12-00522-s001.zip › Supplementary Methods.pdf]

Supporting information

Capturing diversity in seed collections: an empirical study of two congeners with contrasting mating systems

Supplementary Methods

Maximum Likelihood inference of selfing rates

The single-locus Method-of-Moments approach of Ritland (2002) estimates the value of selfing rate, *s*, that maximizes the likelihood of the observed genotypes (of mother and progeny) and population allele frequencies (*p*). To calculate values of $\hat{s}$, we used Equations 14 and 15 from Ritland (2002). We calculated population allele frequencies, *p*, as a simple average across all the individuals (mothers and progeny) in the corresponding population. We then tested our implementation of the estimator by generating simulated progeny genotypes (see below) with three different prescribed selfing rates (s equals 0, 0.5 and 1, respectively). We applied our implementation of the estimator to the simulated data, and in general inferred selfing rates similar to those used in the corresponding simulations (Fig. i), suggesting that this approach was suitable for use with our data. Note that for a given progeny sample, this approach ignores any locus that is missing data for the population allele frequency, or the material or progeny genotype.

Simulations

We performed simulations that generated a set of genotypes at N_L loci for a progeny individual, given a set of population allele frequencies, a set of maternal genotypes, and a prescribed level of selfing (and outcrossing). In general, the maternal genotypes could be drawn from the empirical dataset, or potentially simulated from allele frequencies, with a prescribed level of excess homozygosity (usually denoted as *F*).

In its simplest form, a selfed individual can be simulated by going through each locus and randomly drawing two alleles (with replacement) from the maternal genotype. An outcrossed individual can be simulated by drawing one allele at random from the mother plant, and another from the population, with probabilities that are proportional to the allele frequencies in the population. We also anticipate that biparental inbreeding is important, and so considered the case where the two parents of the progeny were relatives. We represented this by drawing one allele at random from the mother plant. For the second progeny allele, we also drew it from the mother plant with probability *k*, or drew it from the population allele frequencies with probability 1-*k*. Therefore, across different values of *k*, we covered a spectrum from *k*=1, where both alleles were drawn from the mother plant, to *k*=0, where the second allele was drawn from the population allele frequencies. At intermediate *k*, there is a higher probability of inheriting a second copy of the maternal allele, intended to represent identity by descent from a shared recent common ancestor. In this way, *k* represents the relatedness of the mother and father plants, from *k*=1 where the mother and father genotypes are identical, to *k*=0, where the father is drawn from the population at large, and has no elevated probability of sharing alleles that are identical by descent.

Genotyping error

Some population genetic analyses are potentially susceptible to bias due to genotyping error. We wanted to examine how genotyping error rates common in SNP studies might affect estimates of selfing rates. We began with the simplifying assumption that most of the genotyping errors in the dataset involved the failure to observe one of the alleles that is present in a (truly) heterozygous genotype (Luca et al. 2011, Bresadola et al. 2020). We therefore concentrated on the proportion of heterozygous genotypes that are miscalled as homozygotes (of either allele, in equal proportions), which we will denote ε.

Supporting information

Capturing diversity in seed collections: an empirical study of two congeners with contrasting mating systems

We used the count of impossible genotypes, where a progeny individual is homozygous for a different allele to a homozygous mother plant, to obtain an estimate of the value of ε. An impossible genotype would consist of mother genotype AA and progeny genotype aa, or vice versa. Where this occurs, we postulate that this was usually because the true genotypes of mother and progeny consisted of one homozygote (AA or aa) and one heterozygote (Aa), and the heterozygous individual was incorrectly called a homozygote. If this were the case, we would expect to see a relationship among mother progeny pairs, between the number of impossible genotypes (N_{imp}), and the observed number of loci that were either mother-heterozygous and progeny-homozygous, or vice versa (N_{HetHom}). The relationship would be given by the expression $N_{imp} = 0.5\ N_{HetHom}\ \varepsilon$. The 0.5 occurs because only half of the errors that change a heterozygous genotype to a homozygous genotype make the genotype impossible.

Based on this expression, and combining data from both species, we estimated the value of ε for these data to be around 0.03, and potentially in the range 0.01-0.05 (Fig. ii). This estimate is reasonably consistent with previous observations for similar genotyping approaches (Luca et al., 2011). We note that this largely ignores a small group of mother–progeny pairs that had unusually high numbers of impossible genotypes. We note that heterozygous sites are usually less frequent than homozygous sites, such that the genotyping error rate across all loci could be an order of magnitude lower than ε.

Simulating genotypes with error

To impose genotyping errors on simulations, we needed to follow two steps. First, we wanted to estimate a 'hypothetical' true set of genotypes for the mother plant, from which the observed genotypes might have been produced, with errors given by ε. To do this, we needed to estimate the likely proportion of the sites in the mother plant that were observed to be homozygous, but that might have been real heterozygous sites miscalled as homozygous, at rate ε. This can be estimated by the expression:

$$\varepsilon^* = \varepsilon \,/\, (1-\varepsilon) \times N_{Het} \,/\, N_{Hom},$$

where N_{Het} and N_{Hom} are the numbers of heterozygous and homozygous genotypes in the mother plant, respectively. We then estimated a hypothetical genotype for the mother plant by converting observed homozygous genotypes to heterozygotes with probability ε^*. We note that this does not represent an estimate for the true genotype of any mother plant; rather, it is a representation of a possible mother plant, given observed genotypes, and a given rate of error. We used this hypothetical mother plant in the simulation of a progeny individual. We next imposed errors on the progeny individual by converting heterozygous genotypes to homozygous genotypes with probability ε. In all cases, when we decided that a heterozygous genotype would become homozygous, we chose the allele to make homozygous at random.

Inference of Selfing Rates with Neural Network Models

Overview

We next examined the application of neural networks to the inference of selfing rates. Our goal was to find an approach for mating system estimation that could be applied to many loci, and that in future might be extended to data with different properties (e.g., ordered markers), or to inferring different properties of mating systems (such as correlated paternity). Recent reports have shown how Convolutional Neural Networks (CNNs) can be highly effective and flexible when applied to suitable genetic data and problems (Flagel et al. 2019). Often, this involves performing simulations to produce data objects that vary in a specific feature (which can be categorical or a continuous value), and that are 'labelled' with the corresponding values of that feature (Fonseca et al. 2021, Perez et al. 2022). The simulated data objects are then used to 'train' a machine learning model. The model can then be used to predict values of the parameter for a set of analogous empirical data objects, resulting in estimates of the parameter. We performed simulations that produced progeny genotypes under different selfing rates. These were used to train models, along with the corresponding population allele frequencies, maternal genotypes, and selfing rate.

Data for training, validation, and prediction

The basic data object provided to CNN models, applicable to a single (real or simulated) progeny individual, can be thought of as a matrix with three rows and N columns. Each column contains values for a specific SNP locus, such that N is the number of loci. The first row contains the frequency of a reference allele in the population. The second row contains the diploid maternal genotype, encoded as the frequency of the reference allele in the mother plant (0, 0.5, or 1.0). The third row contains the diploid genotypes of the progeny individual, again encoded as the frequency of the reference allele (0, 0.5, or 1.0).

We experimented with different approaches to simulation and training of machine learning models, with three main goals. First, we wanted to produce models that were accurate, based on predictions for 'test' samples, which were simulated in the same way as training data, but not used in the training of models. Second, we wanted to find an approach that was as generally applicable as possible. That is, we began by training separate models for each family (i.e., a model trained for each mother tree), allowing for the possibility that models might only be very narrowly applicable. Later, we relaxed this assumption, and found that we could obtain reasonable performance if we trained a model using simulated progeny for all the families in a dataset, and applied this model to all empirical progeny samples. This substantially reduced the computational effort required to infer selfing rates for all the progeny in each dataset. Third, we explored different model architectures (models), with the goal of using the simplest architecture that produced convincing predictions for an independent set of simulated test data (i.e., extra simulations, not used for training).

For the final analyses, we implemented a CNN with three Conv2D convolutional layers, three Dense layers, and a final regression layer (with 'linear' activation). This regression layer meant the label associated with each data object (progeny individual) was a numerical value (the value of k, representing the amount of selfing used to generate the progeny). For each species, we trained the CNN using 10,000 simulated progeny as training data, and a further 1000 simulated progeny were used as 'validation' data in model training. These validation data are used to estimate loss during training and to adjust training hyper-parameters accordingly. The simulations used a mother plant drawn at random from the data and the allele frequencies of the corresponding population. The simulations used values of k (which controls the level of selfing) drawn from a uniform distribution bounded by 0 and 1. The analyses presented here used values for ε drawn from a uniform distribution between 0.04 and 0.05. We repeated the analyses for very small ε and found that this did not meaningfully affect the outcomes of the analysis. We also tested different schemes for handling missing data in these analyses. This included removing all loci that were missing data for any sample. However, this was quite restrictive to the size of the dataset. Here, for a given progeny individual,

where data were missing for the corresponding population allele frequency or for a genotype (maternal or progeny), we inserted an arbitrary combination of these data that should be equally likely under all models of selfing or outcrossing. This represented a way to make missing data uninformative for the particular combination of sample and locus, and to avoid excluding the locus across all samples. We note that these choices in relation to the specification of models and handling of training data are worthy avenues for future investigation.

We tested the performance of the CNN model by simulating 100 additional progeny for each mother plant using random values of *k*. For each of these simulated progeny datasets, we made a prediction of the selfing rate using the trained CNN. This resulted in 100 'expected' and 'predicted' data points. These are plotted for several exemplar individuals in Fig. iii, and show that, in general, there was strong agreement between predicted and expected selfing rates (Fig. iii). We used these test simulations to calculate a Mean Squared Error (MSE) for each mother plant. These MSE values are potentially a useful index of model performance for different individuals.

Finally, we compared the selfing rates inferred using the Method-of-Moments estimator (MME) approach (Ritland 2002) and the Convolutional Neural Network (CNN) approach. These were strongly concordant among samples (Fig. iv). Both approaches also generate values that serve as an index of confidence in estimated selfing rates, and these also exhibited concordance. For example, in *Hakea sericea* population PT, it was likely inherently difficult to estimate selfing rates accurately due to low heterozygosity. This population had a low value of Average Reciprocal Variance (MME) and a large value of Mean Squared Error (CNN) (Fig. S2).

Supporting information

Capturing diversity in seed collections: an empirical study of two congeners with contrasting mating systems

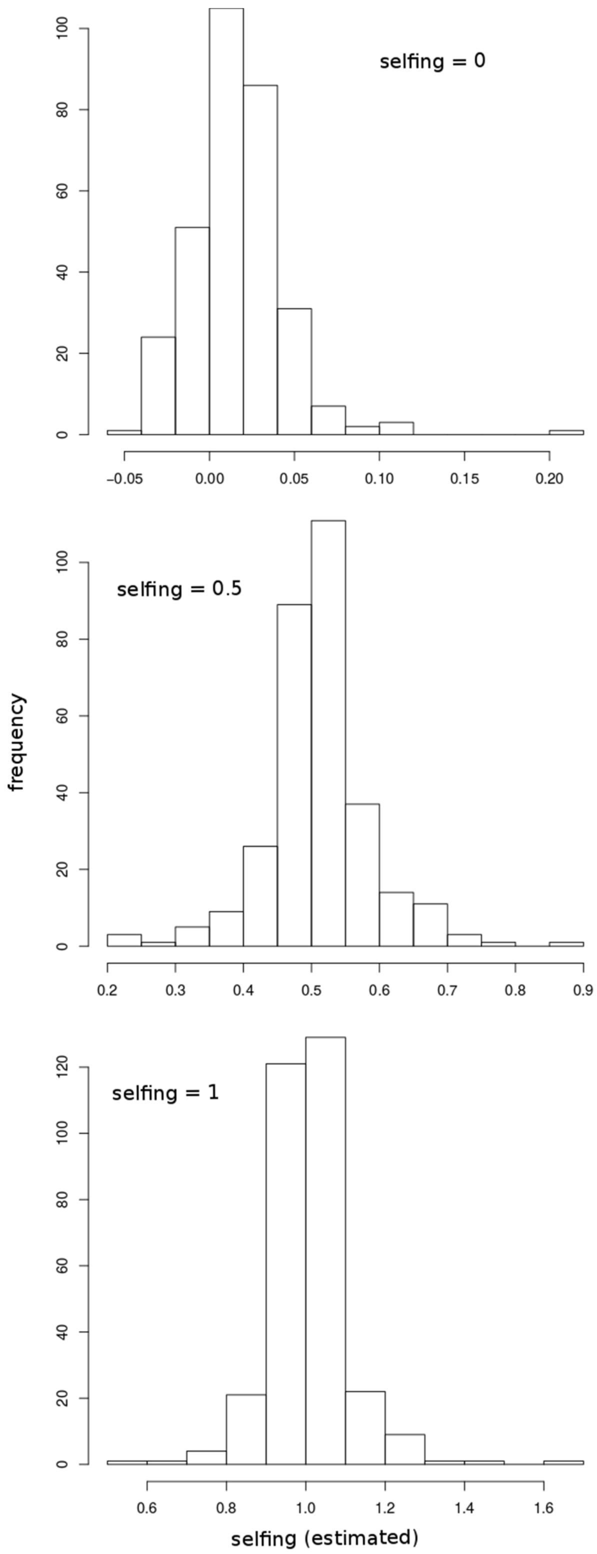

Figure i. Accuracy of the method-of-moments estimator of the single-locus selfing rate. Progeny data were simulated using selfing rates of 0, 0.5, and 1, and the single-locus estimator was used to estimate selfing rate from the simulated data.

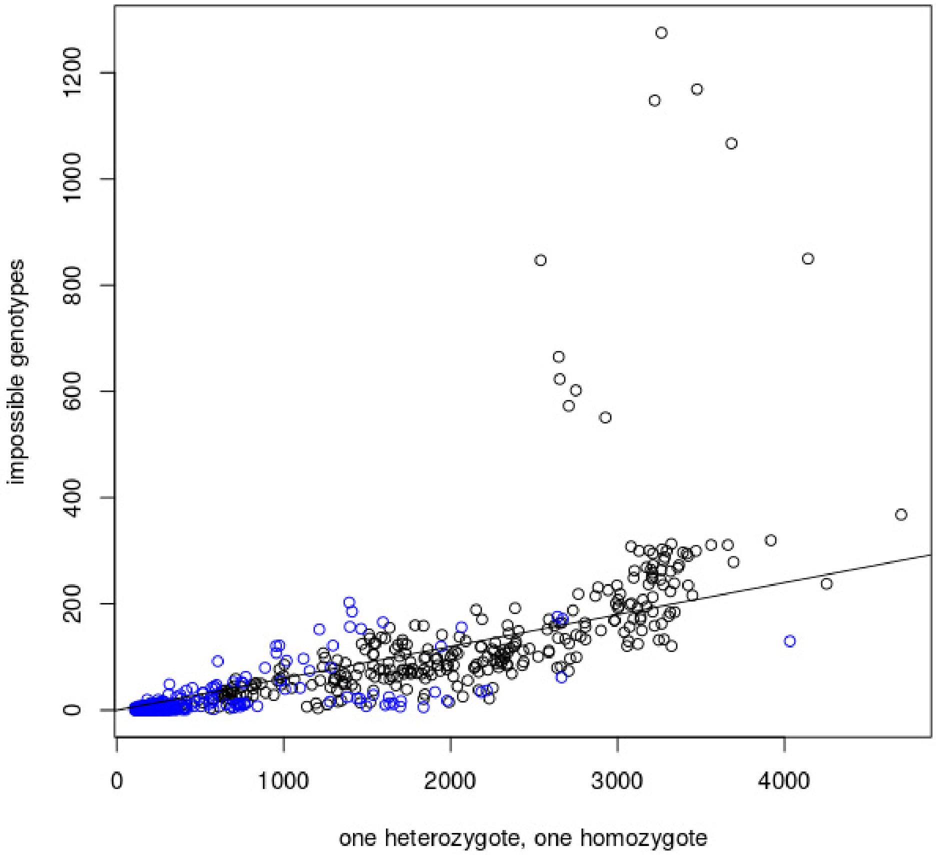

Figure ii. The prevalence of impossible genotypes, where mother and progeny are homozygous for different alleles. For each progeny–mother sample pair, the number of loci with impossible genotypes is shown as a function of the number of loci where one individual (mother or progeny) is heterozygous and the other is homozygous. Black points are *Hakea teretifolia* samples and blue points are *Hakea sericea* samples. The trend line is fit through the origin and has a slope of 0.06 (corresponding to $\varepsilon \approx 0.03$).

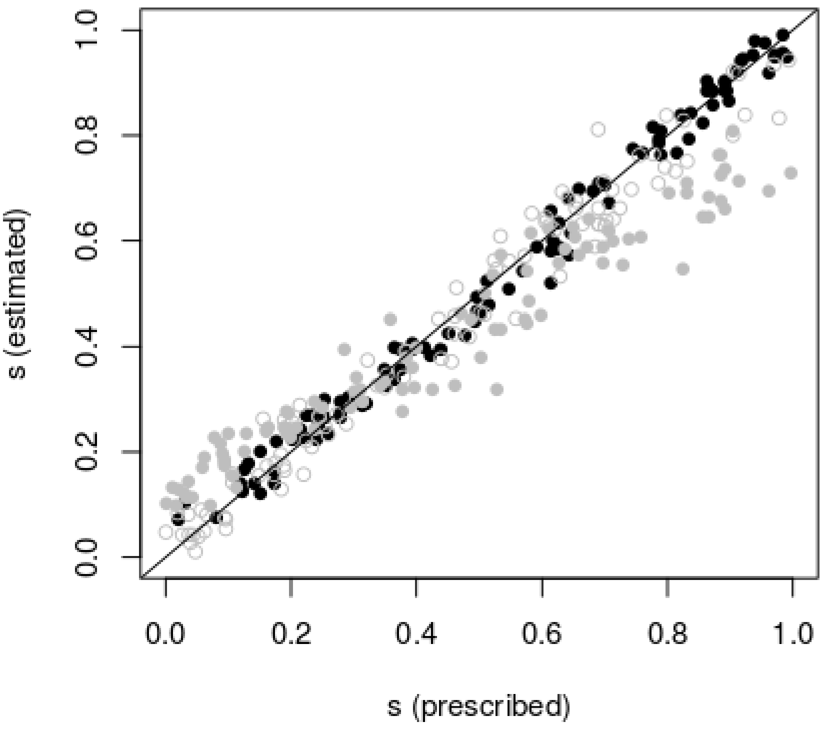

Figure iii. Exemplar validation data for the CNN model. A model was trained to predict selfing rate from genotype data. For each mother plant, 100 datasets were simulated at random levels of selfing. Predictions were made for each dataset. Here, for the mother plant where the model was most (black, filled) and least (gray, filled) accurate, selfing rates predicted using the model are shown as a function of the prescribed (simulated values). For the individual where the predictions were least accurate, a new model was trained using training data and simulations based exclusively on material genotypes (and population allele frequencies) from that individual. Predicted and prescribed points from that model are plotted in unfilled gray symbols.

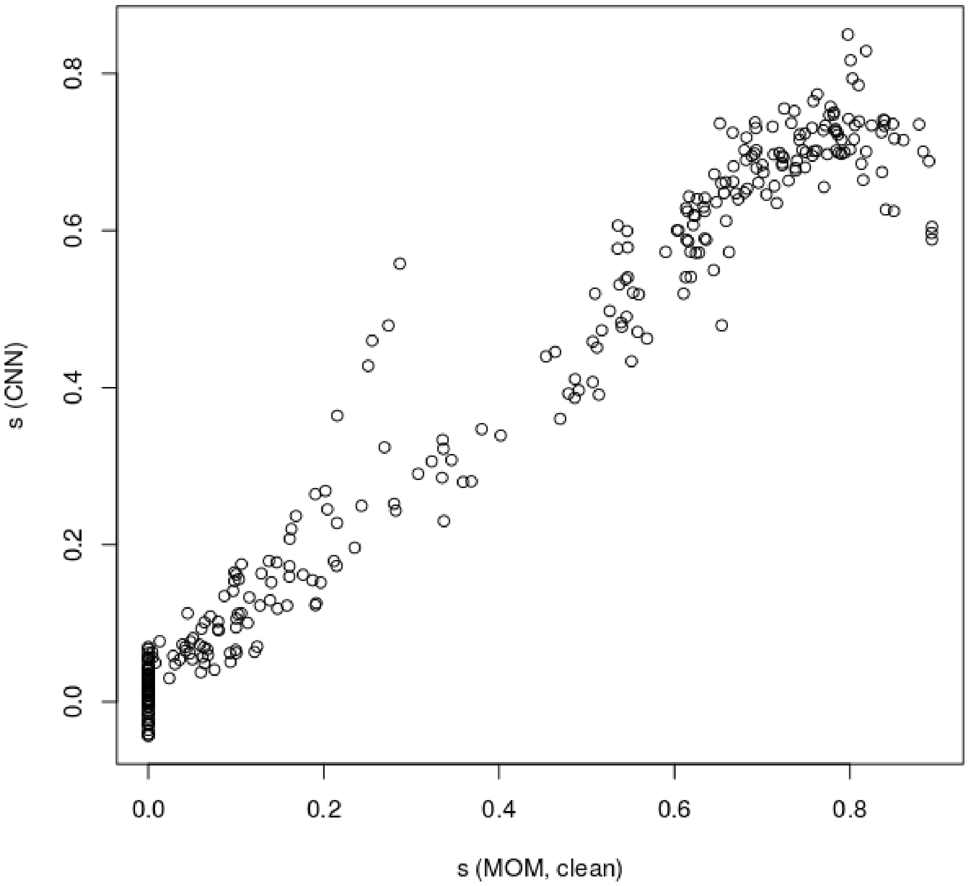

Figure iv. Comparison of selfing-rate estimates from a single-locus method-of-moments (MOM) estimator (horizontal axis) and a CNN model (vertical axis). Each point represents a Hakea sericea progeny individual. Note: estimated values that were < 0 or > 1 have been placed at 0 and 1, respectively, for the purpose of this illustration.
