# Supplementary material for: Capturing Genetic Diversity in Seed Collections: An Empirical Study of Two Congeners with Contrasting Mating Systems
Source: Plants (Basel). 2023 Jan 23;12(3):522. doi: 10.3390/plants12030522 (PMC9921034; doi:10.3390/plants12030522)
Supplement: Supplementary file 1 [file plants-12-00522-s001.zip › Supp_Tables_Figs.pdf]

Supporting information

Capturing diversity in seed collections: an empirical study of two congeners with contrasting mating systems

Table S1. Sample collection sites and details for (a) landscape-scale population genetics (Restore & Renew) sampling; (b) seed (progeny array) sampling.

(a)

species	site	latitude	longitude	date	n samples	type
H. sericea	BlueMountainsNP	-28.5569	153.418	17/10/2015	5	leaf
H. sericea	BrisbaneWatersNP	-33.682	151.1514	19/05/2015	6	leaf
H. sericea	S10	-35.0225	150.4717	23/07/2016	6	leaf
H. sericea	S106	-34.3251	150.4746	6/05/2016	6	leaf
H. sericea	S17	-34.5213	150.2129	21/07/2016	6	leaf
H. sericea	S247	-34.0105	151.2234	11/05/2016	6	leaf
H. sericea	S255	-33.0066	151.4684	13/04/2017	6	leaf
H. sericea	S261	-35.0412	150.1355	13/04/2017	6	leaf
H. sericea	S27	-33.7839	150.6132	18/03/2016	6	leaf
H. sericea	S315	-33.4152	151.004	9/05/2017	6	leaf
H. sericea	S324	-33.9803	151.0084	10/11/2016	6	leaf
H. sericea	S37	-33.3141	150.2451	17/09/2015	6	leaf
H. sericea	S402	-32.7319	152.1305	23/05/2017	6	leaf
H. sericea	S41	-32.8967	150.7616	16/08/2016	6	leaf
H. sericea	S453	-33.4848	151.1868	26/05/2017	6	leaf
H. sericea	S46	-33.1449	151.0966	11/11/2015	6	leaf
H. sericea	S486	-33.5241	150.5658	24/05/2017	6	leaf
H. sericea	S50	-33.6836	150.7614	18/08/2016	6	leaf
H. sericea	S546	-29.9343	152.989	22/06/2017	6	leaf
H. sericea	S56	-34.1382	150.9983	31/08/2016	5	leaf
H. sericea	S57	-33.4765	150.7773	30/12/2016	5	leaf
H. sericea	S585	-35.1281	150.0123	10/07/2017	6	leaf
H. sericea	S612	-33.6635	150.6113	13/07/2017	6	leaf
H. sericea	S67	-32.8831	151.4473	21/04/2016	5	leaf
H. sericea	S68	-34.3519	150.631	8/09/2016	6	leaf
H. sericea	S73	-33.7848	150.3967	21/04/2016	6	leaf
H. teretifolia	BlueMountainsNP	-33.6737	151.2086	11/06/2015	6	leaf
H. teretifolia	BrisbaneWatersNP	-33.6717	151.1371	19/05/2015	6	leaf
H. teretifolia	HeathcoteNP	-33.5516	151.2954	18/03/2016	6	leaf
H. teretifolia	DuffysForest	-33.6737	151.2086	20/05/2016	5	leaf
H. teretifolia	JervisBayNP	-32.7454	152.1131	12/04/2016	6	leaf
H. teretifolia	HeathcoteNP	-33.8168	151.3005	14/10/2015	7	leaf
H. teretifolia	RoyalNP	-35.1072	150.6784	25/07/2015	6	leaf
H. teretifolia	S156	-35.1123	150.3161	15/03/2017	6	leaf
H. teretifolia	S162	-32.1167	152.4441	16/03/2017	6	leaf
H. teretifolia	S177	-33.6244	151.2606	28/03/2017	6	leaf
H. teretifolia	S18	-32.5015	152.2875	21/11/2016	6	leaf

H. teretifolia	S19	-33.5406	151.2825	3/09/2015	6	leaf
H. teretifolia	S21	-34.1693	150.9951	14/03/2016	6	leaf
H. teretifolia	S31	-31.3136	152.8723	12/04/2016	6	leaf
H. teretifolia	S324	-31.7608	152.745	10/11/2016	5	leaf
H. teretifolia	S34	-34.1155	150.9491	13/04/2016	6	leaf
H. teretifolia	S43	-33.7953	150.4044	19/04/2016	6	leaf
H. teretifolia	S47	-33.6556	151.2568	18/08/2016	6	leaf
H. teretifolia	S55	-33.5879	150.2676	20/04/2016	6	leaf
H. teretifolia	S56	-35.0225	150.4717	31/08/2016	5	leaf
H. teretifolia	S588	-33.6078	150.3253	10/07/2017	6	leaf
H. teretifolia	S591	-34.6731	150.7168	10/07/2017	6	leaf
H. teretifolia	S594	-34.138	150.9971	10/07/2017	6	leaf
H. teretifolia	S60	-33.165	151.5796	4/01/2017	6	leaf
H. teretifolia	S600	-34.1404	151.1156	11/07/2017	6	leaf
H. teretifolia	S603	-34.2048	150.8464	11/07/2017	6	leaf
H. teretifolia	S73	-33.5768	150.3634	21/04/2016	6	leaf
H. teretifolia	S8	-33.4472	151.2139	28/06/2016	6	leaf

(b)

species	site	latitude	longitude	date	n samples	type
H. sericea	BV	-33.6461	151.1045	28/10/2018	10	leaf, seed
H. sericea	CR	-34.9776	150.738	13/12/2018	10	leaf, seed
H. sericea	KL	-34.0124	151.2244	12/10/2018	10	leaf, seed
H. sericea	MO	-33.4869	151.1873	7/12/2018	10	leaf, seed
H. sericea	PT	-32.8967	150.7613	29/11/2018	10	leaf, seed
H. sericea	SP	-33.6861	150.5779	28/10/2018	11	leaf, seed
H. teretifolia	BV	-33.6438	151.1141	28/10/2018	10	leaf, seed
H. teretifolia	CR	-35.0015	150.7927	13/12/2018	10	leaf, seed
H. teretifolia	KL	-34.0131	151.2251	12/10/2018	10	leaf, seed
H. teretifolia	MB	-33.5782	150.3635	26/10/2018	10	leaf, seed
H. teretifolia	MU	-33.1649	151.5792	7/12/2018	10	leaf, seed
H. teretifolia	RN	-34.1137	151.0677	22/11/2018	10	leaf, seed
H. teretifolia	VC	-33.5879	150.2677	26/10/2018	10	leaf, seed

Supporting information
Capturing diversity in seed collections: an empirical study of two congeners with contrasting mating systems

Table S2. Seed (progeny array) details and germination results.

species	site	mat. line	n sown	n germ.	% germ.	n sequenced
H. sericea	BV	1	15	15	100	3
H. sericea	BV	2	18	15	83	3
H. sericea	BV	3	10	10	100	3
H. sericea	BV	4	18	16	89	12
H. sericea	BV	5	11	11	100	5
H. sericea	BV	6	26	21	81	10
H. sericea	BV	7	6	6	100	1
H. sericea	BV	8	12	12	100	3
H. sericea	BV	9	15	15	100	10
H. sericea	BV	10	12	12	100	3
H. sericea	CR	1	20	18	90	3
H. sericea	CR	2	16	16	100	3
H. sericea	CR	3	24	24	100	3
H. sericea	CR	4	26	26	100	12
H. sericea	CR	5	26	24	92	3
H. sericea	CR	6	22	19	86	3
H. sericea	CR	7	22	22	100	3
H. sericea	CR	8	24	24	100	10
H. sericea	CR	9	22	22	100	3
H. sericea	CR	10	13	12	92	10
H. sericea	KL	1	20	20	100	10
H. sericea	KL	2	20	19	95	3
H. sericea	KL	3	20	20	100	9
H. sericea	KL	4	17	17	100	3
H. sericea	KL	5	19	12	63	0
H. sericea	KL	6	19	19	100	3
H. sericea	KL	7	20	20	100	3
H. sericea	KL	8	19	19	100	3
H. sericea	KL	9	20	20	100	10
H. sericea	KL	10	20	20	100	3
H. sericea	MO	1	24	23	96	10
H. sericea	MO	2	24	24	100	9
H. sericea	MO	3	12	11	92	4
H. sericea	MO	4	6	5	83	2
H. sericea	MO	5	6	6	100	3
H. sericea	MO	6	18	17	94	3
H. sericea	MO	7	8	8	100	3
H. sericea	MO	8	12	12	100	3
H. sericea	MO	9	16	14	88	9
H. sericea	MO	10	8	7	88	3
H. sericea	PT	1	24	24	100	10
H. sericea	PT	2	24	23	96	12
H. sericea	PT	3	24	18	75	3
H. sericea	PT	4	24	17	71	3
H. sericea	PT	5	22	18	82	3
H. sericea	PT	6	24	24	100	10

Supporting information
Capturing diversity in seed collections: an empirical study of two congeners with contrasting mating systems

H. sericea	PT	7	21	17	81	3
H. sericea	PT	8	18	17	94	3
H. sericea	PT	9	23	18	78	3
H. sericea	PT	10	22	19	86	3
H. sericea	SP	1	18	13	72	10
H. sericea	SP	2	10	10	100	3
H. sericea	SP	3	20	20	100	12
H. sericea	SP	4	12	11	92	3
H. sericea	SP	5	8	8	100	3
H. sericea	SP	6	6	6	100	3
H. sericea	SP	7	6	6	100	3
H. sericea	SP	8	17	13	76	10
H. sericea	SP	9	12	12	100	3
H. sericea	SP	10	10	10	100	3
H. sericea	SP	11	12	12	100	3
H. teretifolia	BV	1	24	20	83	10
H. teretifolia	BV	2	19	11	58	1
H. teretifolia	BV	3	15	7	47	3
H. teretifolia	BV	4	14	7	50	3
H. teretifolia	BV	5	7	1	14	0
H. teretifolia	BV	6	24	11	46	6
H. teretifolia	BV	7	24	9	38	3
H. teretifolia	BV	8	14	4	29	3
H. teretifolia	BV	9	4	2	50	2
H. teretifolia	BV	10	24	20	83	9
H. teretifolia	CR	1	24	23	96	9
H. teretifolia	CR	2	24	19	79	0
H. teretifolia	CR	3	25	25	100	3
H. teretifolia	CR	4	21	17	81	3
H. teretifolia	CR	5	24	24	100	10
H. teretifolia	CR	6	28	22	79	3
H. teretifolia	CR	7	24	13	54	3
H. teretifolia	CR	8	25	23	92	10
H. teretifolia	CR	9	28	27	96	3
H. teretifolia	CR	10	28	25	89	3
H. teretifolia	KL	1	6	5	83	2
H. teretifolia	KL	2	9	8	89	3
H. teretifolia	KL	3	14	4	29	3
H. teretifolia	KL	4	24	16	67	10
H. teretifolia	KL	5	16	15	94	9
H. teretifolia	KL	6	18	9	50	3
H. teretifolia	KL	7	24	21	88	10
H. teretifolia	KL	8	21	11	52	3
H. teretifolia	KL	9	3	2	67	3
H. teretifolia	KL	10	17	7	41	3
H. teretifolia	MB	1	25	14	56	9
H. teretifolia	MB	2	12	2	17	1
H. teretifolia	MB	3	15	4	27	3
H. teretifolia	MB	4	24	21	88	10

Supporting information

Capturing diversity in seed collections: an empirical study of two congeners with contrasting mating systems

H. teretifolia	MB	5	16	16	100	3
H. teretifolia	MB	6	23	14	61	3
H. teretifolia	MB	7	18	10	56	3
H. teretifolia	MB	8	10	8	80	3
H. teretifolia	MB	9	24	23	96	10
H. teretifolia	MB	10	25	15	60	3
H. teretifolia	MU	1	22	3	14	3
H. teretifolia	MU	2	24	7	29	5
H. teretifolia	MU	3	25	5	20	6
H. teretifolia	MU	4	19	4	21	4
H. teretifolia	MU	5	22	5	23	3
H. teretifolia	MU	6	24	1	4	1
H. teretifolia	MU	7	16	5	31	5
H. teretifolia	MU	8	24	4	17	5
H. teretifolia	MU	9	23	4	17	3
H. teretifolia	MU	10	21	3	14	5
H. teretifolia	RN	1	20	5	25	3
H. teretifolia	RN	2	26	20	77	3
H. teretifolia	RN	3	24	18	75	10
H. teretifolia	RN	4	24	22	92	3
H. teretifolia	RN	5	28	23	82	10
H. teretifolia	RN	6	24	17	71	3
H. teretifolia	RN	7	23	12	52	3
H. teretifolia	RN	8	24	24	100	4
H. teretifolia	RN	9	24	4	17	2
H. teretifolia	RN	10	18	17	94	10
H. teretifolia	VC	1	16	4	25	3
H. teretifolia	VC	2	24	1	4	0
H. teretifolia	VC	3	16	1	6	0
H. teretifolia	VC	4	25	0	0	0
H. teretifolia	VC	5	18	7	39	7
H. teretifolia	VC	6	17	8	47	8
H. teretifolia	VC	7	13	5	38	2
H. teretifolia	VC	8	16	3	19	2
H. teretifolia	VC	9	24	5	21	4
H. teretifolia	VC	10	13	1	8	1

Table S3. Estimates of genetic diversity for seed sampling sites (per-site Ho, He, FIS, relatedness, average genetic distance from mother plants; average genetic distance from all individuals).

species	site	Ho	He	FIS	average pairwise relatedness	average pairwise Euclidean distance (mums)	average pairwise Euclidean distance (all)
H. sericea	BV	0.094	0.193	0.424	0.059	88.249	85.790
H. sericea	CR	0.026	0.103	0.699	0.157	62.173	48.169
H. sericea	KL	0.021	0.143	0.823	0.128	72.603	68.620
H. sericea	MO	0.057	0.1	0.365	0.068	59.600	56.775
H. sericea	PT	0.018	0.013	-0.149	0.381	15.693	15.356
H. sericea	SP	0.015	0.094	0.84	0.265	54.486	63.033
H. teretifolia	BV	0.120	0.191	0.311	0.052	99.253	93.421
H. teretifolia	CR	0.081	0.173	0.436	0.073	98.387	93.814
H. teretifolia	KL	0.139	0.195	0.228	0.056	95.900	92.135
H. teretifolia	MB	0.134	0.191	0.241	0.056	95.920	96.160
H. teretifolia	MU	0.135	0.142	0.036	0.053	72.956	75.511
H. teretifolia	RN	0.175	0.201	0.100	0.057	93.658	98.384
H. teretifolia	VC	0.102	0.160	0.290	0.071	85.262	85.624

Supporting information

Capturing diversity in seed collections: an empirical study of two congeners with contrasting mating systems

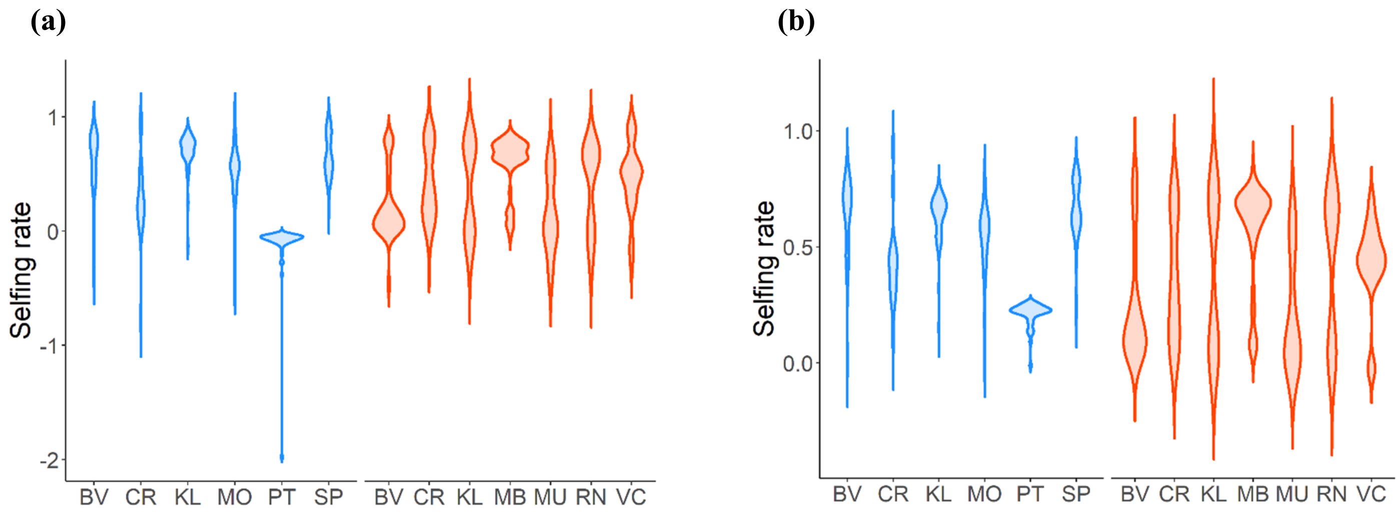

Figure S1. Violin plots (Gaussian kernel density estimates) of individual progeny selfing rates inferred per site using (a) MME and (b) CNN approaches for *H. sericea* (blue) and *H. teretifolia* (orange). Estimates for all sites are shown, including outlier estimates from site PT at which all mother plants were highly genetically homogeneous.

Supporting information

Capturing diversity in seed collections: an empirical study of two congeners with contrasting mating systems

(a) **(b)**

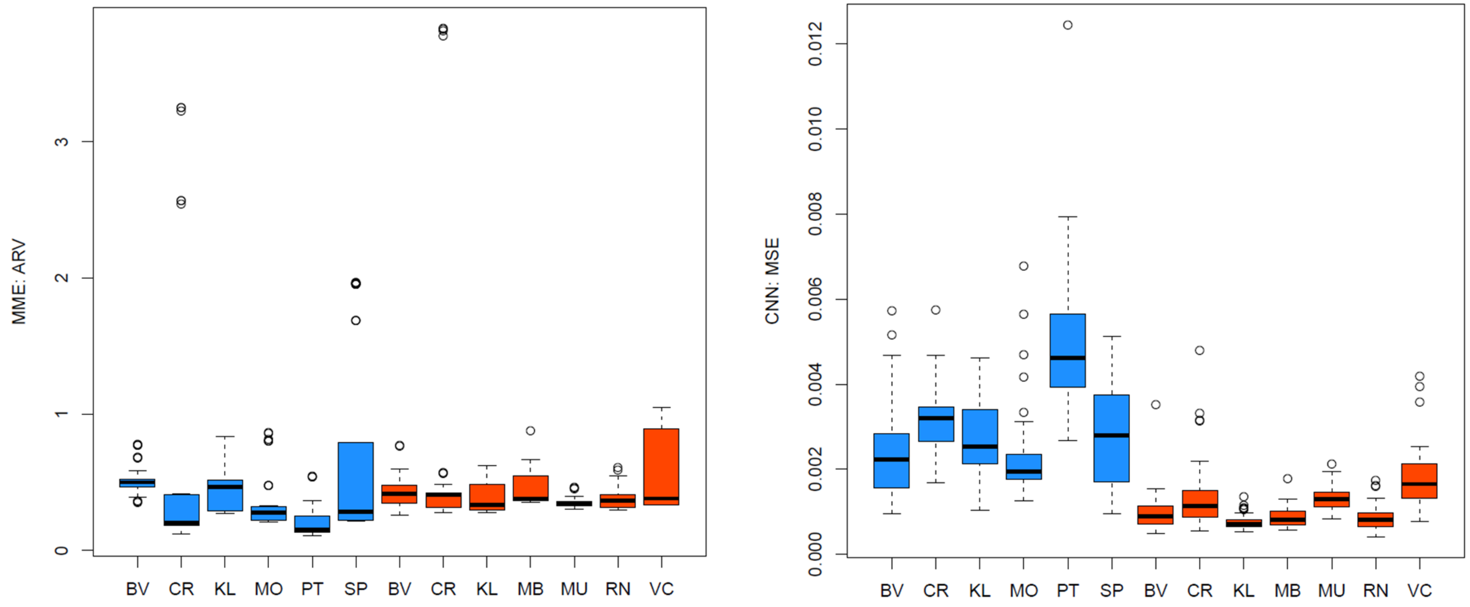

Figure S2. Measures of (a) average reciprocal variance among loci calculated as part of MME inference of selfing rate, and (b) mean standard error from CNN estimation of selfing rate among progeny at each site in *H. sericea* (blue) and *H. teretifolia* (orange). Less informative data for estimation of selfing rates are expected to result in lower ARV and higher MSE values relative to more informative data. Thus, less confidence might be warranted in estimates with relatively low ARV and/or relatively high MSE.

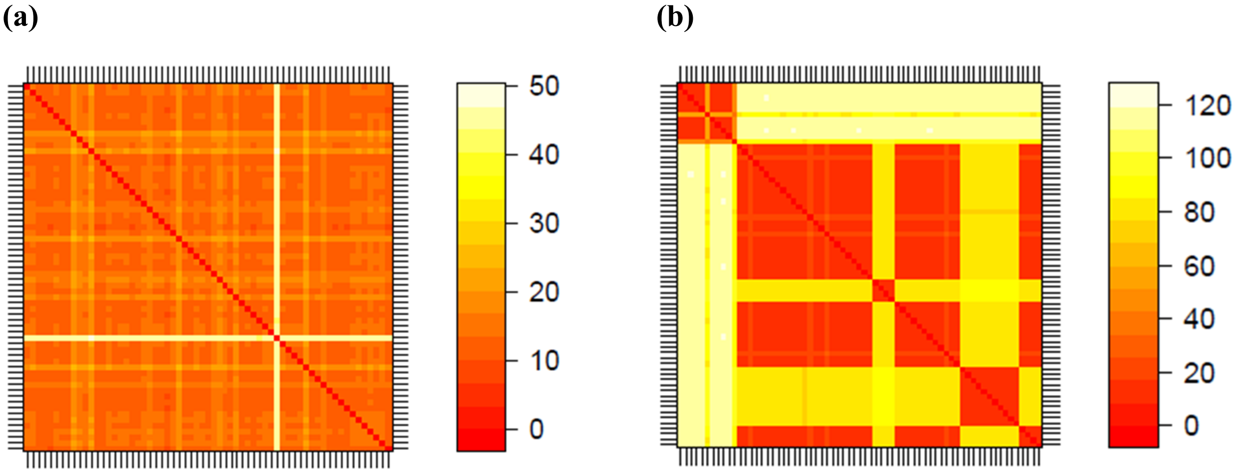

Figure S3. Heatmaps of pairwise Euclidean distance matrices among *H. sericea* individuals originating from (a) site PT and (b) site SP (at which observed heterozygosity is similar to PT). Matrices are ordered by maternal line (mother followed by offspring). High similarity among all individuals across maternal lines at site PT is indicated by near-uniformly low pairwise genetic distance (orange-red tones). In contrast, four distinct groups of similar individuals are discernible at site SP, corresponding to maternal lines (with the largest of these groups comprising 8 of the 11 maternal lines sampled at this site).

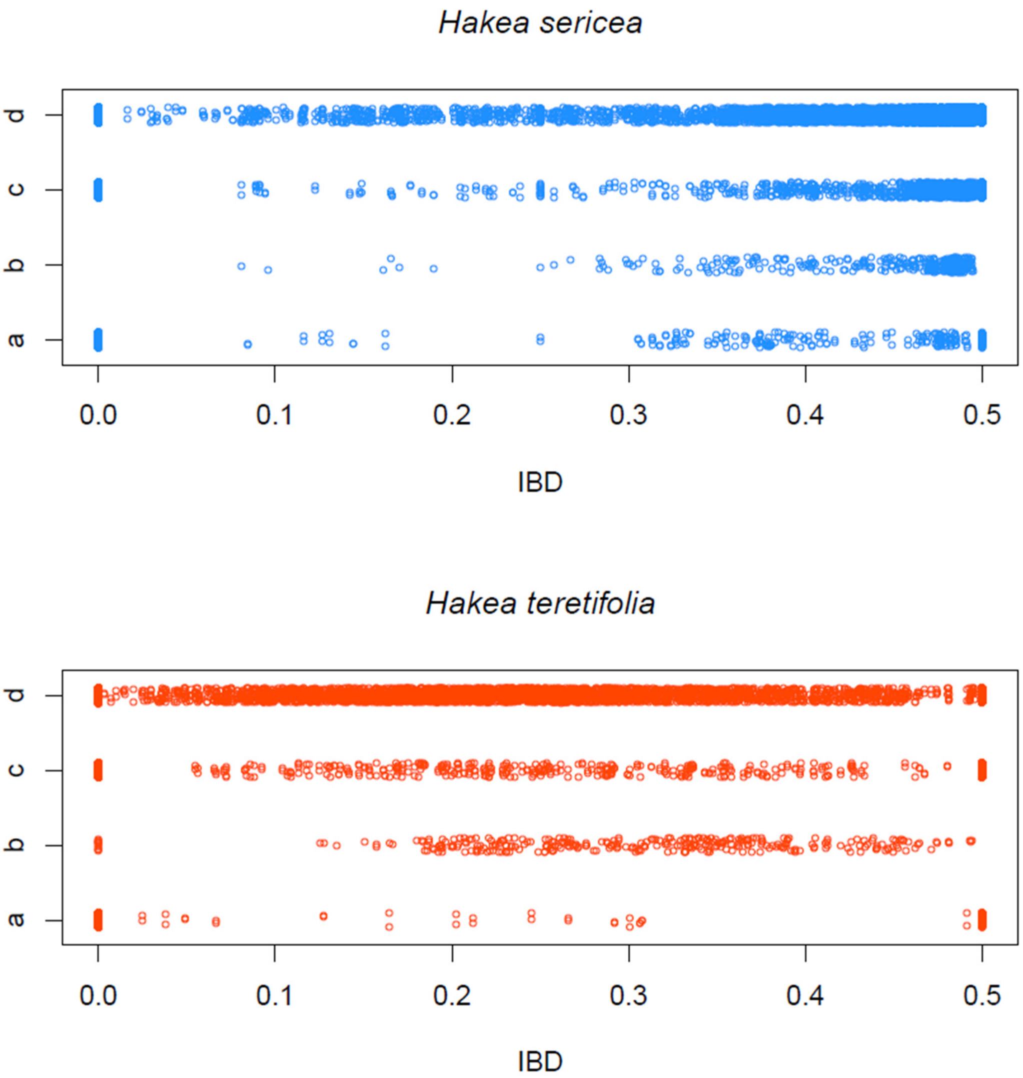

Figure S4. Stripcharts plotting pairwise kinship estimates among (a) mothers within populations, (b) mothers and offspring, (c) siblings within populations, (d) all individuals within populations. IBD= identity by descent. On average, pairwise kinship coefficients among mothers within populations were significantly higher in *H. sericea* (0.18, vs. 0.059 in *H. teretifolia*, $p = 6.2 \times 10^{-27}$), as were parent-progeny kinship coefficients (*H. sericea*: 0.44, *H. teretifolia*: 0.30, $p = 2.6 \times 10^{-58}$), and kinship coefficients among siblings (*H. sericea*: 0.27, *H. teretifolia*: 0.12, $p = 8.0 \times 10^{-69}$).

Supporting information

Capturing diversity in seed collections: an empirical study of two congeners with contrasting mating systems

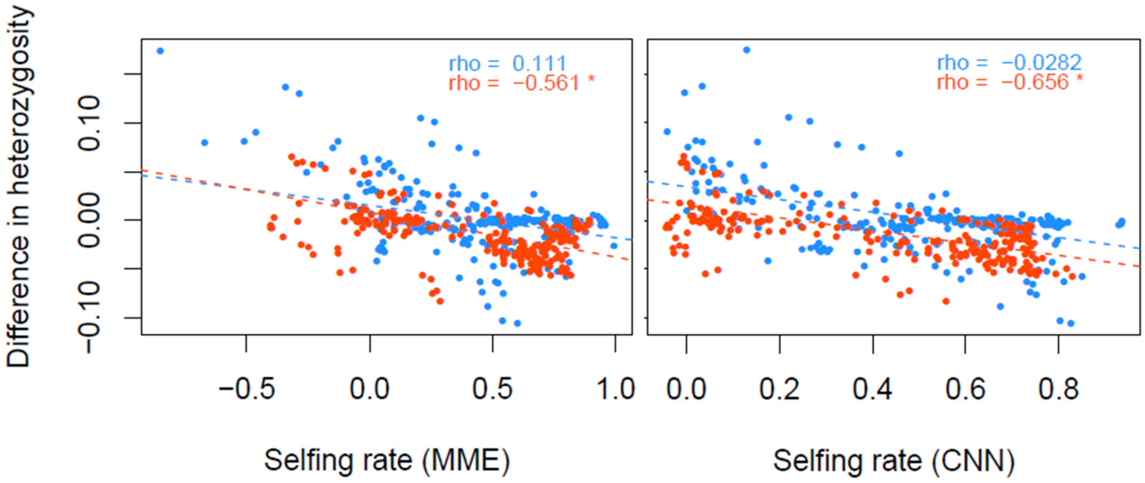

Figure S5. Difference in proportion of heterozygous loci between individual seedlings and their mothers, plotted against each seedling's estimated selfing rate (*H. sericea*: blue; *H. teretifolia*: orange). Shown for each species: linear regression (dashed) lines; Spearman's correlation coefficient (rho), asterisks denoting significant correlation ($p < 0.05$).

Supporting information

Capturing diversity in seed collections: an empirical study of two congeners with contrasting mating systems

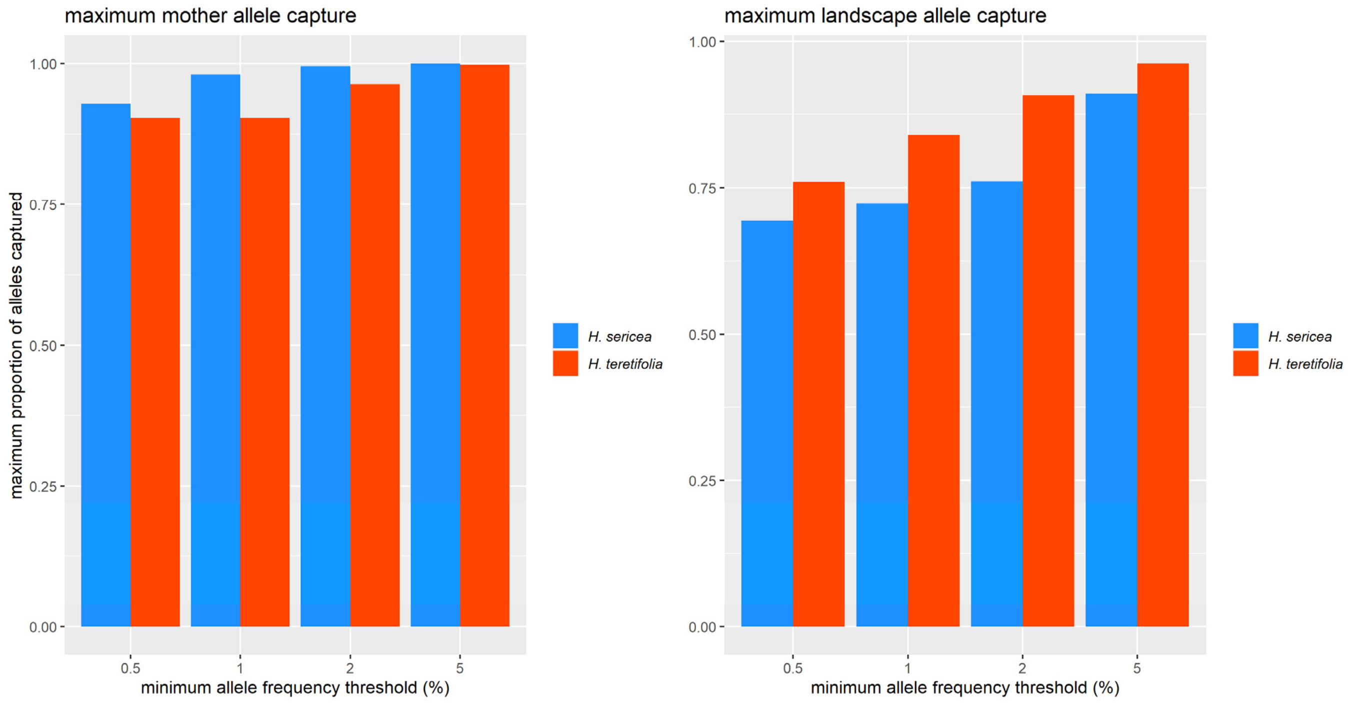

Figure S6. Maximum proportions of alleles captured by resampling seedlings (8 maternal lines among all seed sampling sites), for (a) alleles present in the mother plants at seed sampling sites, and (b) alleles present in all adult plants including the landscape-scale sampling sites. Maximum allele capture is shown for alleles present in each data set at four different frequencies (i.e., allele frequency >= 0.5%, 1%, 2%, and 5%).
